# Supplementary material for: Nickel(II) Coordination Polymers Supported by Bis-pyridyl-bis-amide and Angular Dicarboxylate Ligands: Role of Ligand Flexibility in Iodine Adsorption
Source: Int J Mol Sci. 2022 Mar 25;23(7):3603. doi: 10.3390/ijms23073603 (PMC8998537; doi:10.3390/ijms23073603)
Supplement: Supplementary file 1 [file ijms-23-03603-s001.zip › ijms-1622222-supplementary.pdf]

Supplementary materials

Nickel(II) Coordination Polymers Supported by Bis-pyridyl-bis-amide and Angular Dicarboxylate Ligands: Synthesis, Structures and Iodine Adsorption

Wei-Te Lee, Tsung-Te Liao and Jhy-Der Chen*

Department of Chemistry, Chung-Yuan Christian University, Chung Li 32023, Taiwan
lku23y230230@gmail.com (W.-T.L.); zxc0927808262@gmail.com (T.-T.L.)

* Correspondence: jdchen@cycu.edu.tw (J.-D.C.); Tel.: +886-3-265-3351

Figure S1. Simulated and experimental PXRD patterns of **1**.

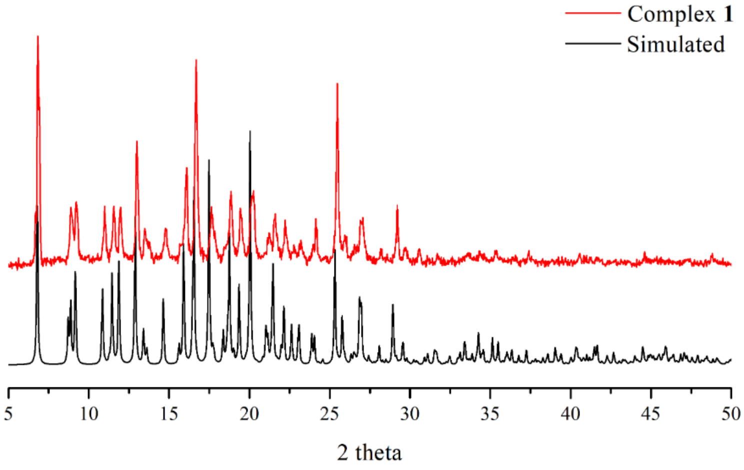

Figure S2. Simulated and experimental PXRD patterns of **2**.

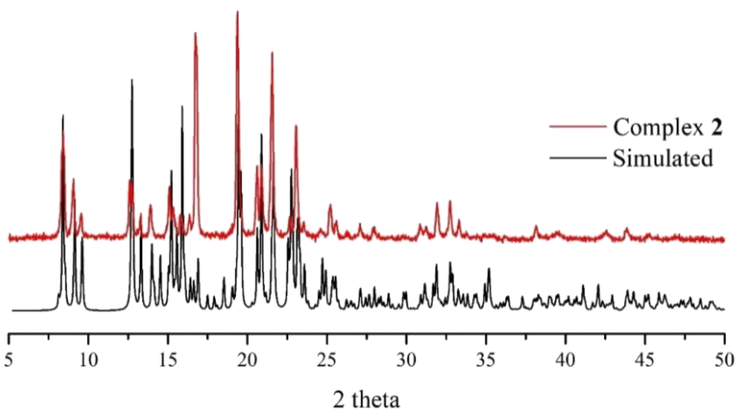

Figure S3. Simulated and experimental PXRD patterns of **3**.

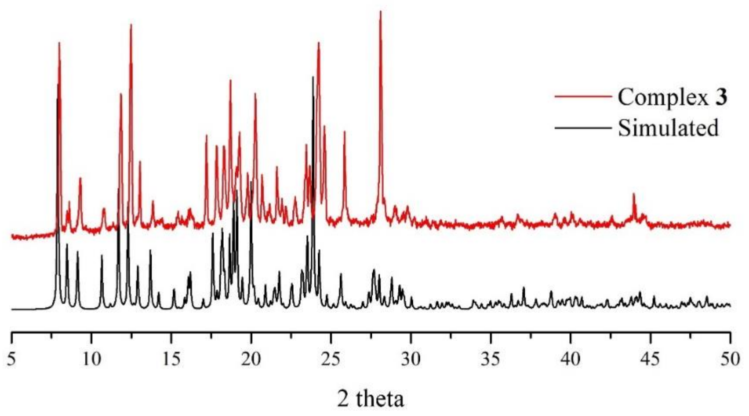

Figure S4. Simulated and experimental PXRD patterns of **4**.

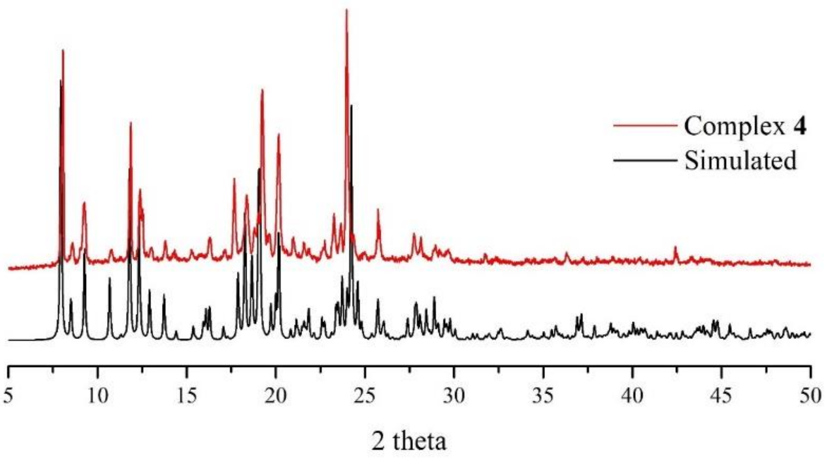

Figure S5. Simulated and experimental PXRD patterns of **5**.

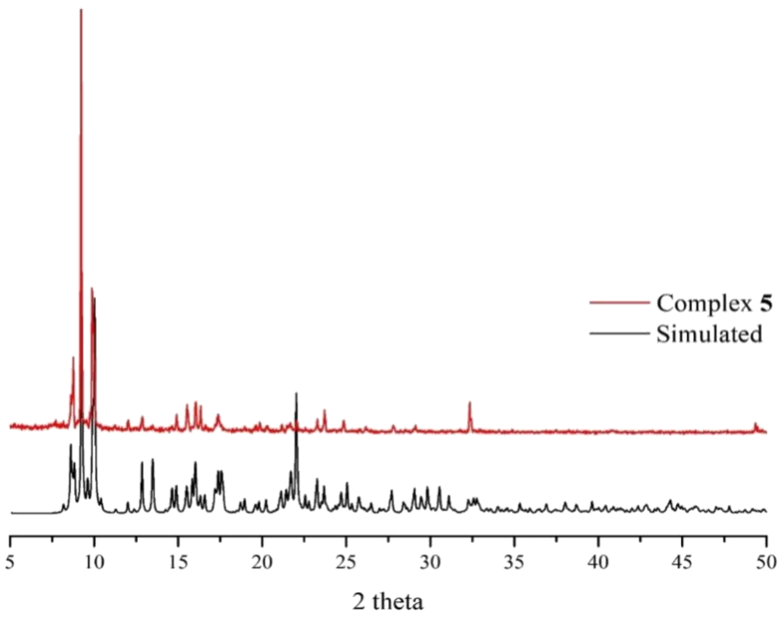

Figure S6. Simulated and experimental PXRD patterns of **6**.

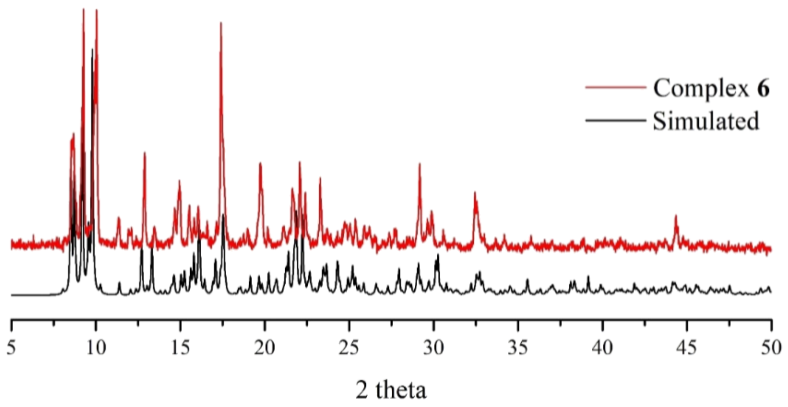

Figure S7. Simulated and experimental PXRD patterns of **7.**

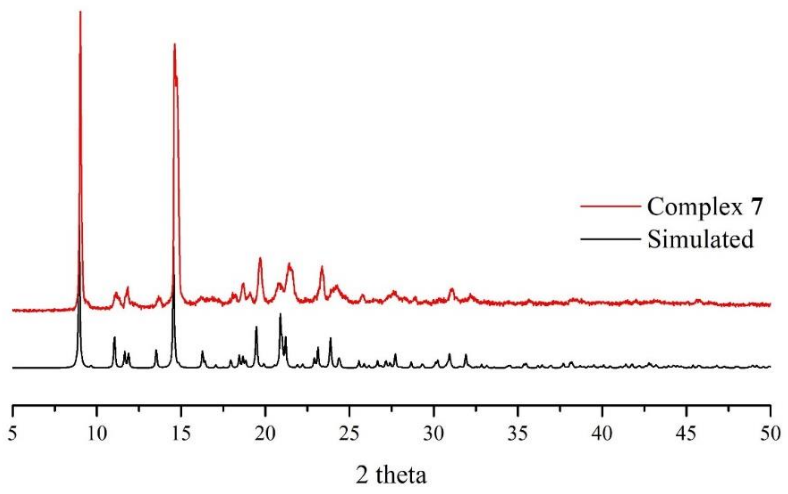

Figure S8. Simulated and experimental PXRD patterns of **8**.

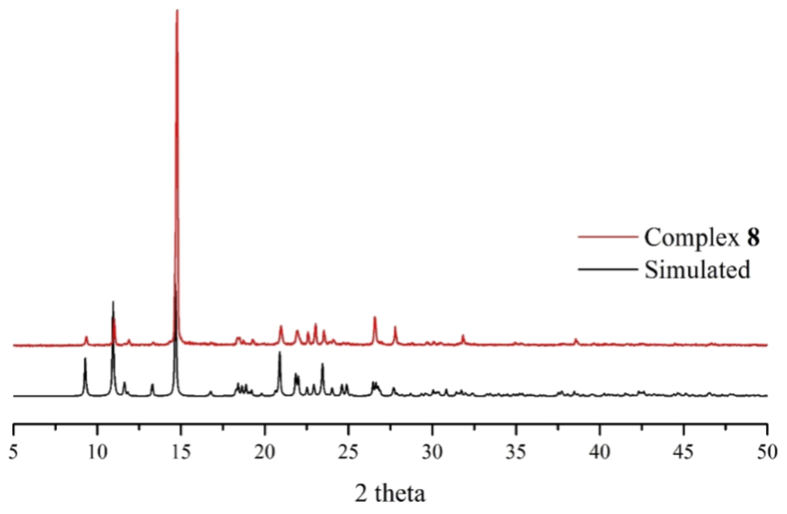

Figure S9. Simulated and experimental PXRD patterns of **9**.

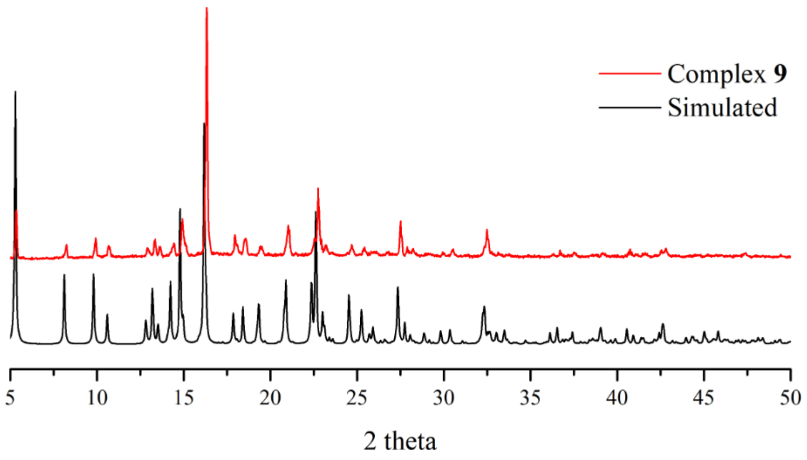

Figure S10. The TGA curve for complex **1**.

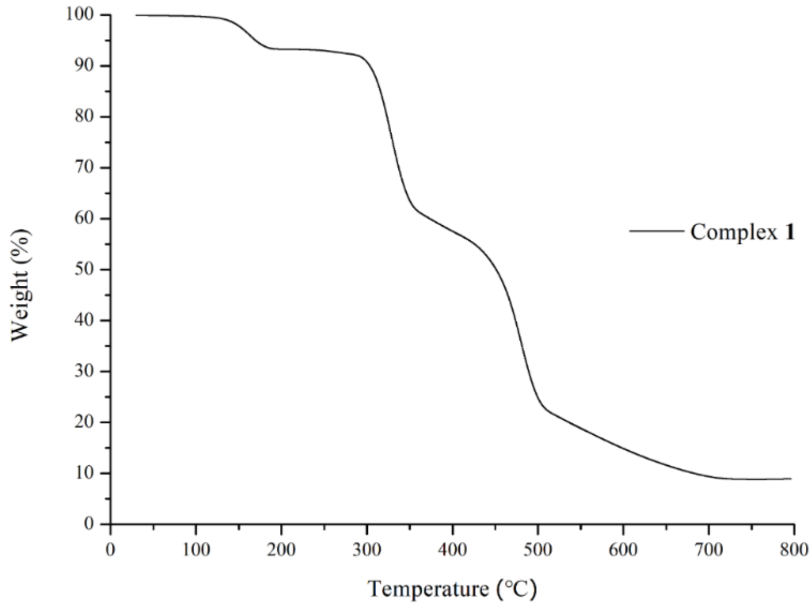

Figure S11. The TGA curve for complex **2**.

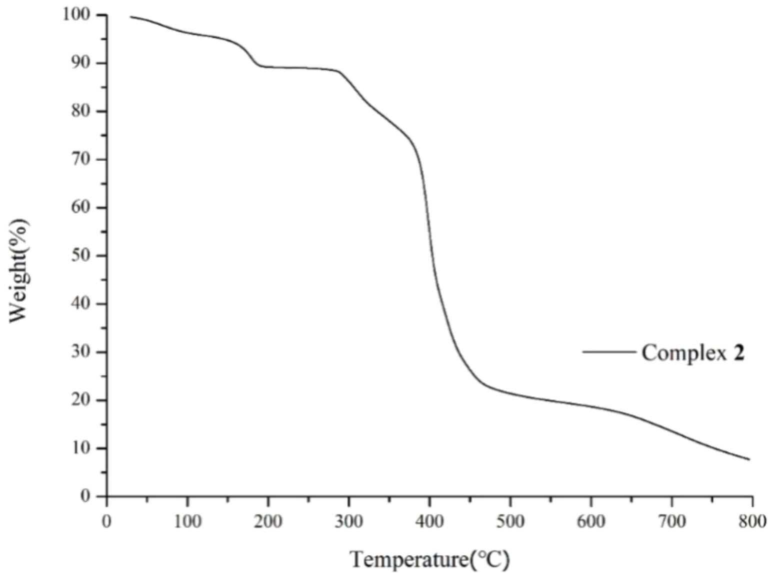

Figure S12. The TGA curve for complex **3**.

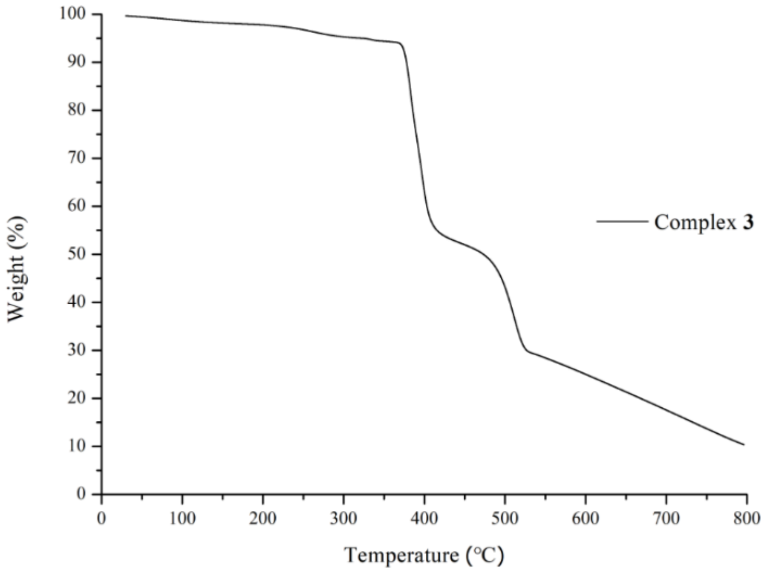

Figure S13. The TGA curve for complex **4**.

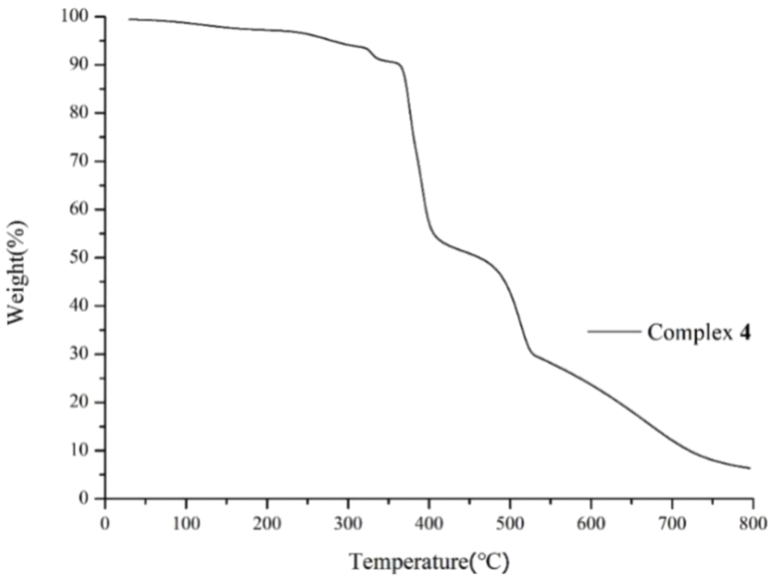

Figure S14. The TGA curve for complex **5**.

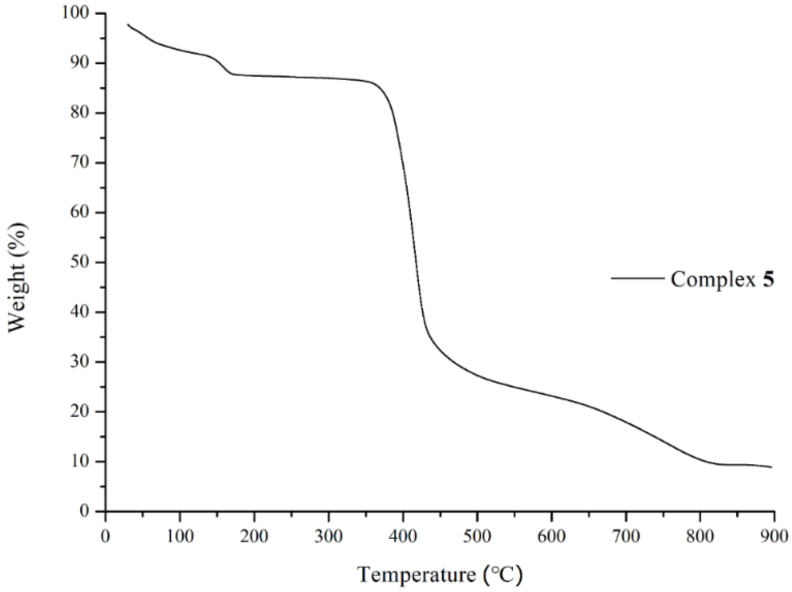

Figure 15. The TGA curve for complex **6**.

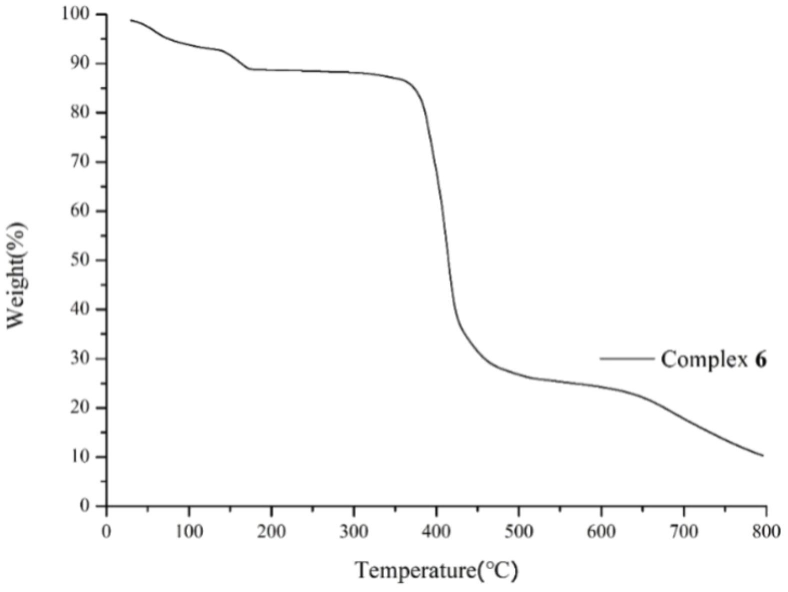

Figure S16. The TGA curve for complex **7**.

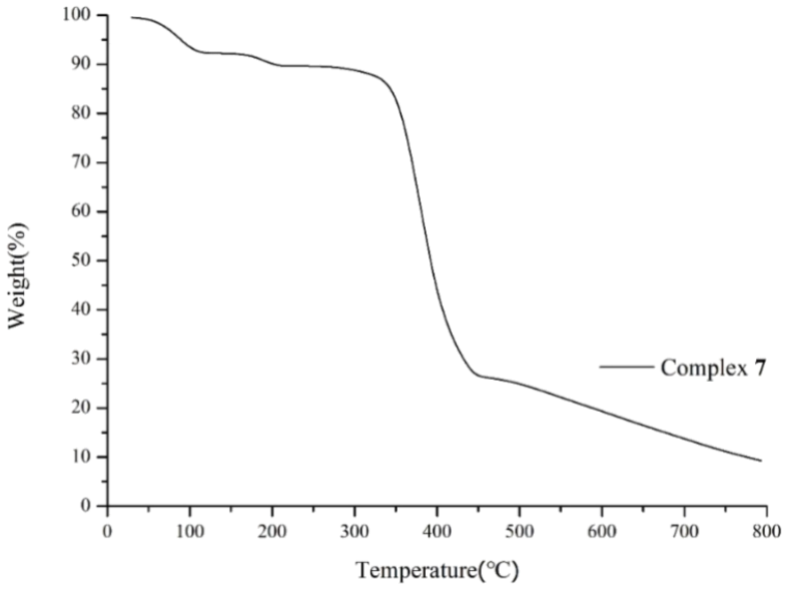

Figure S17. The TGA curve for complex **8**.

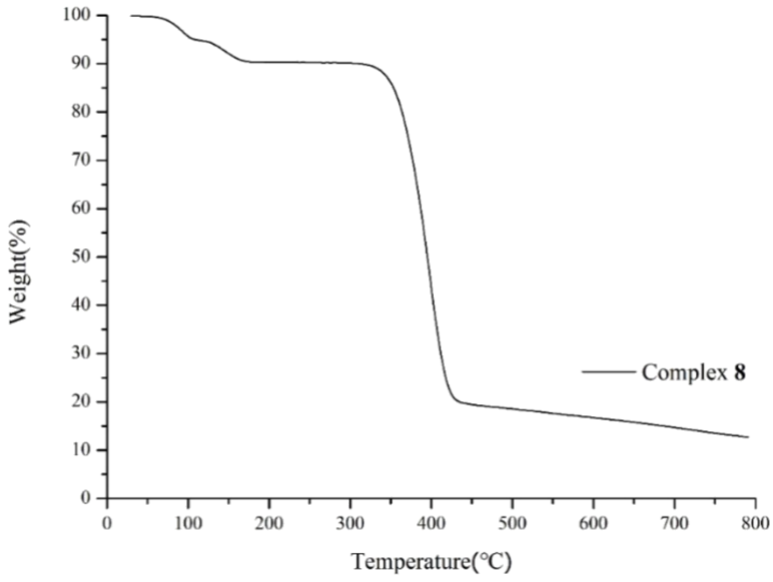

Figure S18. The TGA curve for complex **9**.

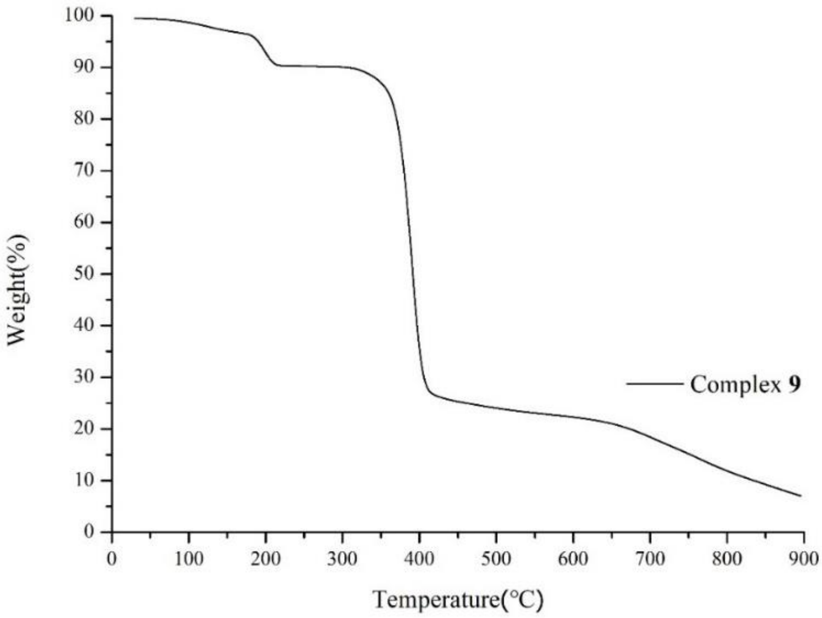

Table S1. Iodine adsorption experiments for complex **7** at 25 °C.

adsorption times (min)	times	initial weight (mg)	finial weight (mg)	Iodine content (mg)	Weight change (mg g^{-1})	average (mg g^{-1})
30	1	35.193	35.201	0.008	0.227	**0.208**
	2	35.225	35.232	0.007	0.199	
	3	35.227	35.232	0.007	0.199	
60	1	35.192	35.206	0.014	0.398	**0.379**
	2	35.215	35.228	0.013	0.369	
	3	35.225	35.238	0.013	0.369	
120	1	35.219	35.237	0.018	0.511	**0.521**
	2	35.216	35.238	0.019	0.540	
	3	35.225	35.243	0.018	0.511	
180	1	35.210	35.290	0.021	0.596	**0.587**
	2	35.222	35.305	0.020	0.568	
	3	35.228	35.249	0.021	0.596	
360	1	35.227	35.249	0.022	0.625	**0.606**
	2	35.225	35.246	0.021	0.596	
	3	35.228	35.249	0.021	0.596	

Table S2. Iodine adsorption experiments for complex **7** at 60 °C.

adsorption times (min)	times	initial weight (mg)	finial weight (mg)	Iodine content (mg)	Weight change (mg g^{-1})	average (mg g^{-1})
	1	35.201	35.215	0.014	0.398	
30	2	35.222	35.235	0.013	0.369	**0.379**
	3	35.221	35.234	0.013	0.369	
	1	35.180	35.199	0.019	0.540	
60	2	35.281	35.301	0.020	0.568	**0.549**
	3	35.262	35.287	0.019	0.540	
	1	35.180	35.201	0.021	0.596	
120	2	35.281	35.301	0.020	0.568	**0.587**
	3	35.262	35.289	0.021	0.596	
	1	35.197	35.219	0.022	0.625	
180	2	35.288	35.309	0.021	0.596	**0.606**
	3	35.268	35.289	0.021	0.596	
	1	35.240	35.262	0.022	0.625	
360	2	35.240	35.261	0.021	0.596	**0.606**
	3	35.262	35.283	0.021	0.596	

Table S3. Iodine adsorption experiments for complex **8** at 25 ºC.

adsorption times (min)	times	initial weight (mg)	finial weight (mg)	Iodine content (mg)	Weight change (mg g^{-1})	average (mg g^{-1})
	1	38.521	38.522	0.001	0.258	
30	2	38.518	38.519	0.001	0.258	**0.258**
	3	38.512	38.513	0.001	0.258	
	1	38.517	38.519	0.002	0.519	
60	2	38.510	38.512	0.002	0.519	**0.606**
	3	38.511	38.514	0.003	0.779	
	1	38.520	38.524	0.004	1.038	
120	2	38.515	38.519	0.004	1.038	**0.952**
	3	38.516	38.519	0.003	0.779	
	1	38.520	38.525	0.005	1.298	
180	2	38.519	38.524	0.005	1.298	**1.298**
	3	38.518	38.523	0.005	1.298	
	1	38.520	38.525	0.005	1.298	
360	2	38.521	38.527	0.006	1.558	**1.385**
	3	38.521	38.527	0.006	1.558	

Table S4. Iodine adsorption experiments for complex **8** at 60 °C.

adsorption times (min)	times	initial weight (mg)	finial weight (mg)	Iodine content (mg)	Weight change (mg g^{-1})	average (mg g^{-1})
	1	38.521	38.523	0.002	0.519	
30	2	38.518	38.520	0.002	0.519	**0.606**
	3	38.512	38.515	0.003	0.779	
	1	38.517	38.522	0.005	1.298	
60	2	38.515	38.520	0.005	1.298	**1.298**
	3	38.511	38.516	0.005	1.298	
	1	38.520	38.526	0.006	1.558	
120	2	38.515	38.521	0.006	1.558	**1.558**
	3	38.516	38.522	0.006	1.558	
	1	38.520	38.526	0.006	1.558	
180	2	38.521	38.527	0.006	1.558	**1.558**
	3	38.522	38.528	0.006	1.558	
	1	38.520	38.526	0.006	1.558	
360	2	38.520	38.526	0.006	1.558	**1.558**
	3	38.521	38.527	0.006	1.558	

Table S5. Iodine adsorption experiments for complex **9** at 25 °C.

adsorption times (min)	try	initial weight (mg)	finial weight (mg)	Iodine content (mg)	Weight change (mg g^{-1})	average (mg g^{-1})
30	1	29.35	29.98	0.63	21.45	**22.73**
	2	29.34	30.02	0.68	23.18	
	3	29.30	29.99	0.69	23.55	
60	1	29.36	31.15	1.79	60.97	**63.72**
	2	29.34	31.33	1.99	67.83	
	3	29.35	31.18	1.83	62.35	
120	1	29.36	32.83	3.47	118.19	**118.93**
	2	29.37	32.88	3.51	119.51	
	3	29.39	32.89	3.50	119.09	
180	1	29.34	34.08	4.74	161.55	**159.75**
	2	29.32	34.01	4.68	159.62	
	3	29.35	33.99	4.64	158.09	
360	1	29.36	34.15	4.79	163.15	**163.14**
	2	29.38	34.18	4.80	163.38	
	3	29.34	34.10	4.78	162.92	

Table S6. Iodine adsorption experiments for complex **9** at 60 °C.

adsorption times (min)	try	initial weight (mg)	finial weight (mg)	Iodine content (mg)	Weight change (mg g^{-1})	average (mg g^{-1})
	1	29.36	32.50	3.14	106.94	
30	2	29.34	32.47	3.13	106.68	**107.92**
	3	29.33	32.56	3.23	110.13	
	1	29.36	33.52	4.16	141.69	
60	2	29.37	33.49	4.12	140.28	**140.64**
	3	29.37	33.58	4.11	139.94	
	1	29.36	34.15	4.79	163.15	
120	2	29.37	34.06	4.69	159.69	**161.30**
	3	29.37	34.12	4.73	161.05	
	1	29.39	34.25	4.86	165.36	
180	2	29.38	34.26	4.88	166.10	**165.15**
	3	29.39	34.21	4.82	164.00	
	1	29.36	34.25	4.89	166.55	
360	2	29.38	34.28	4.90	166.78	**166.55**
	3	29.34	34.20	4.88	166.33	

Figure S19. Color changes of complex **7** heated at 25 and 60 ^{o}C: (a) without heating and heated for (b) 30, (c) 60, (d) 120, (e) 180 and (f) 360 minutes.

25 ^{o}C

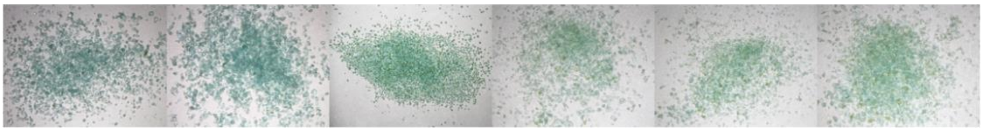

(a) (b) (c) (d) (e) (f)

60 ^{o}C

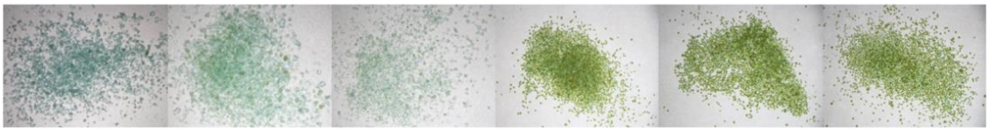

(a) (b) (c) (d) (e) (f)

Figure S20. Color changes of complex **8** heated at 25 and 60 ^{o}C: (a) without heating and heated for (b) 30, (c) 60, (d) 120, (e) 180 and (f) 360 minutes.

25 ^{o}C

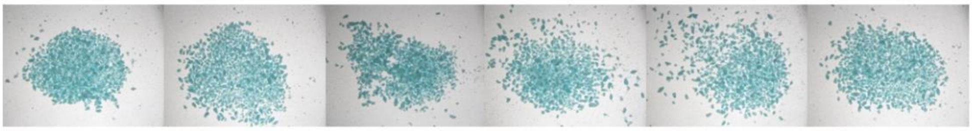

(a) (b) (c) (d) (e) (f)

60 ^{o}C

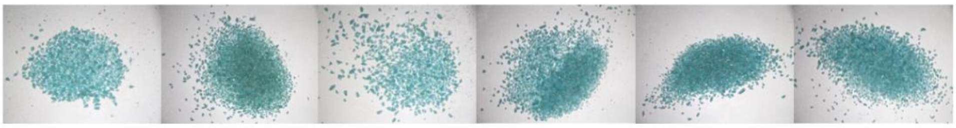

(a) (b) (c) (d) (e) (f)

Figure S21. Color changes of complex **9** heated at 25 and 60 °C: (a) without heating and heated for (b) 30, (c) 60, (d) 120, (e) 180 and (f) 360 minutes.

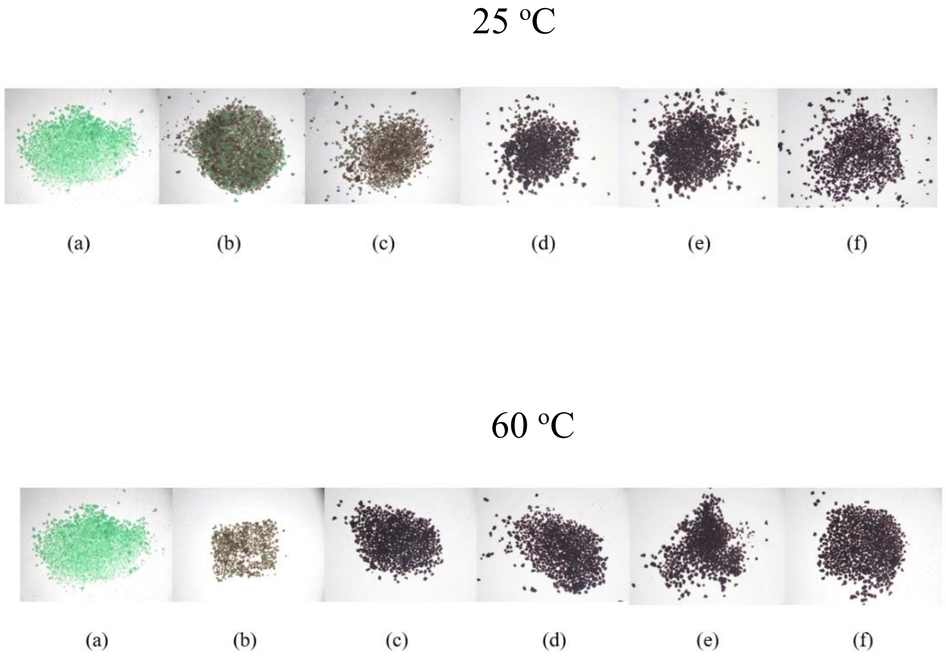

Figure S22. PXRD patterns of complex **7** after heated at (a) 25 and (b) 60 °C for various intervals.

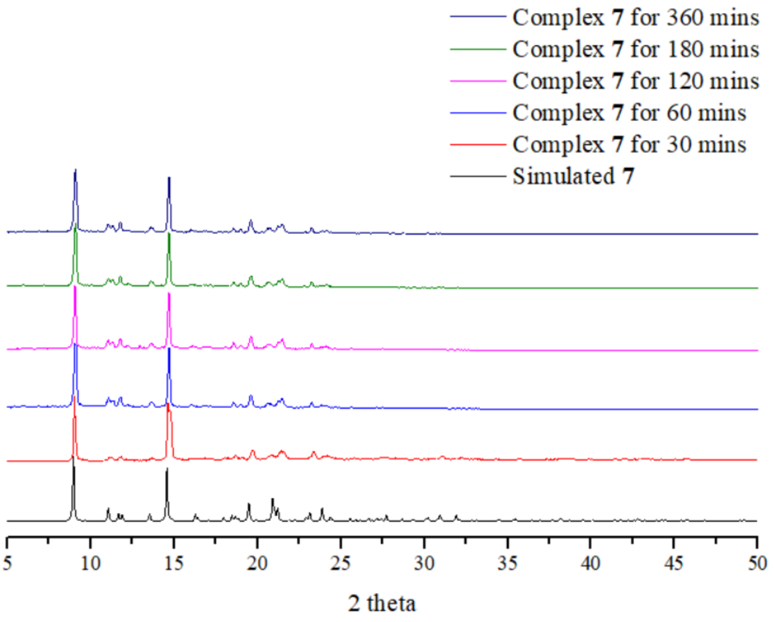

(a)

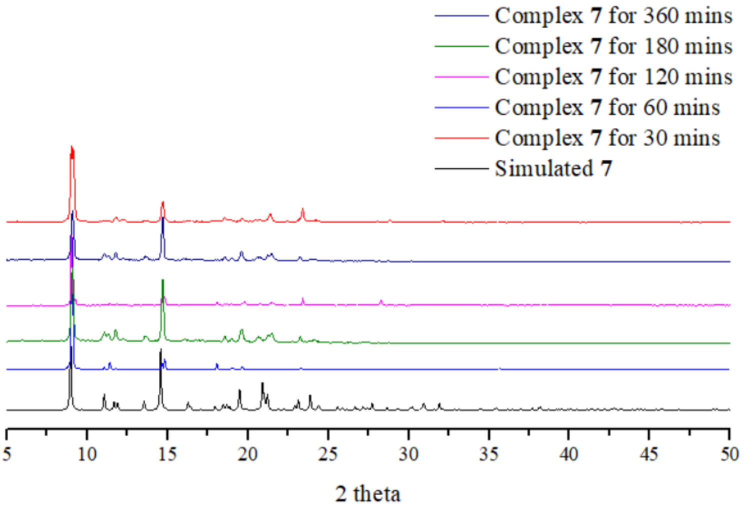

(b)

Figure S23. PXRD patterns of complex **8** after heated at (a) 25 and (b) 60 °C for various intervals.

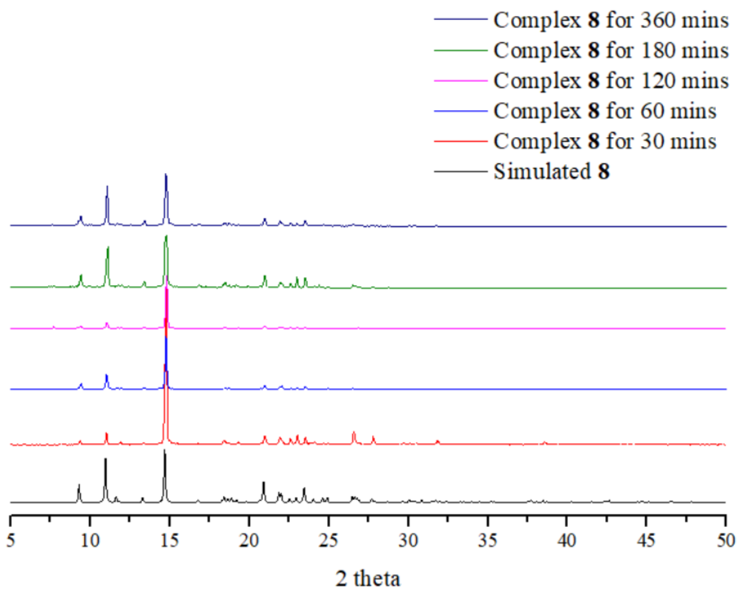

(a)

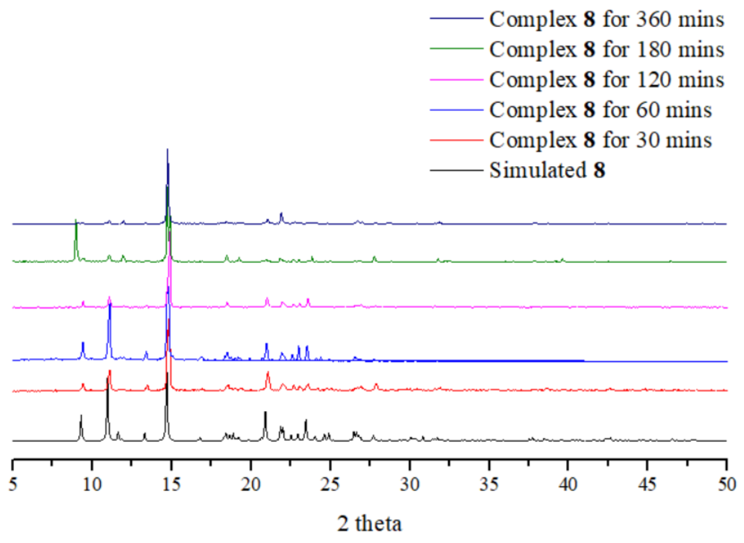

(b)

Figure S24. PXRD patterns of complex **9** after heated at (a) 25 and (b) 60 °C for various intervals.

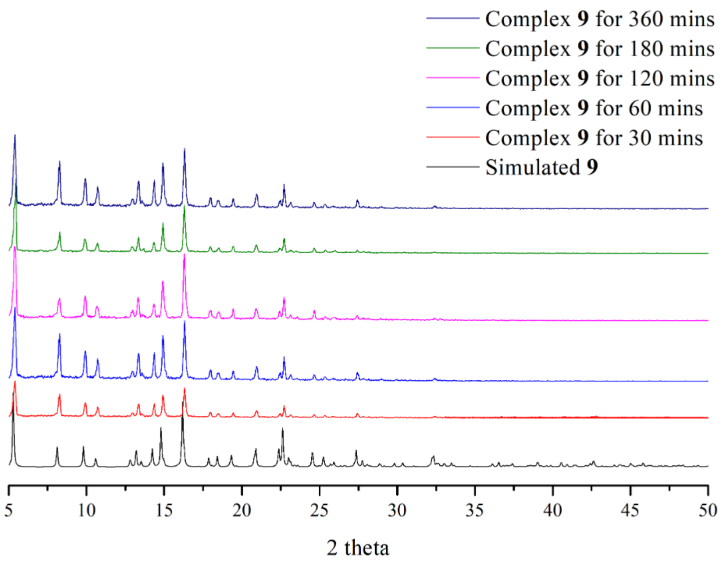

(a)

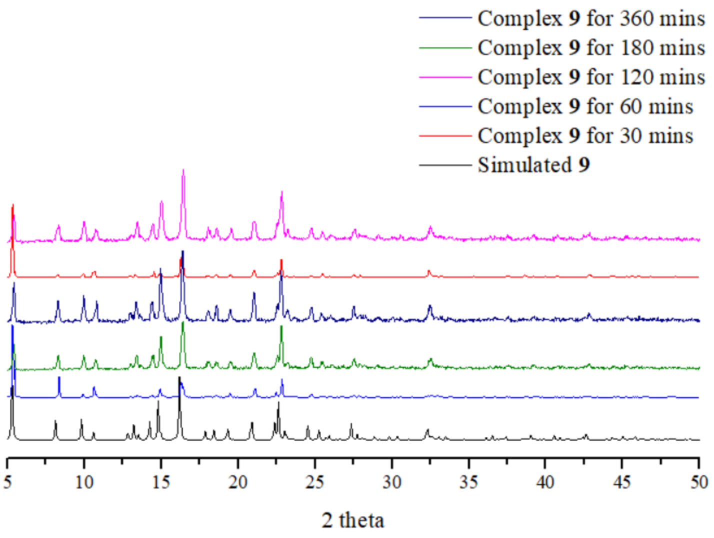

(b)

Figure S25. EDX data for complex **7** at three different regions.

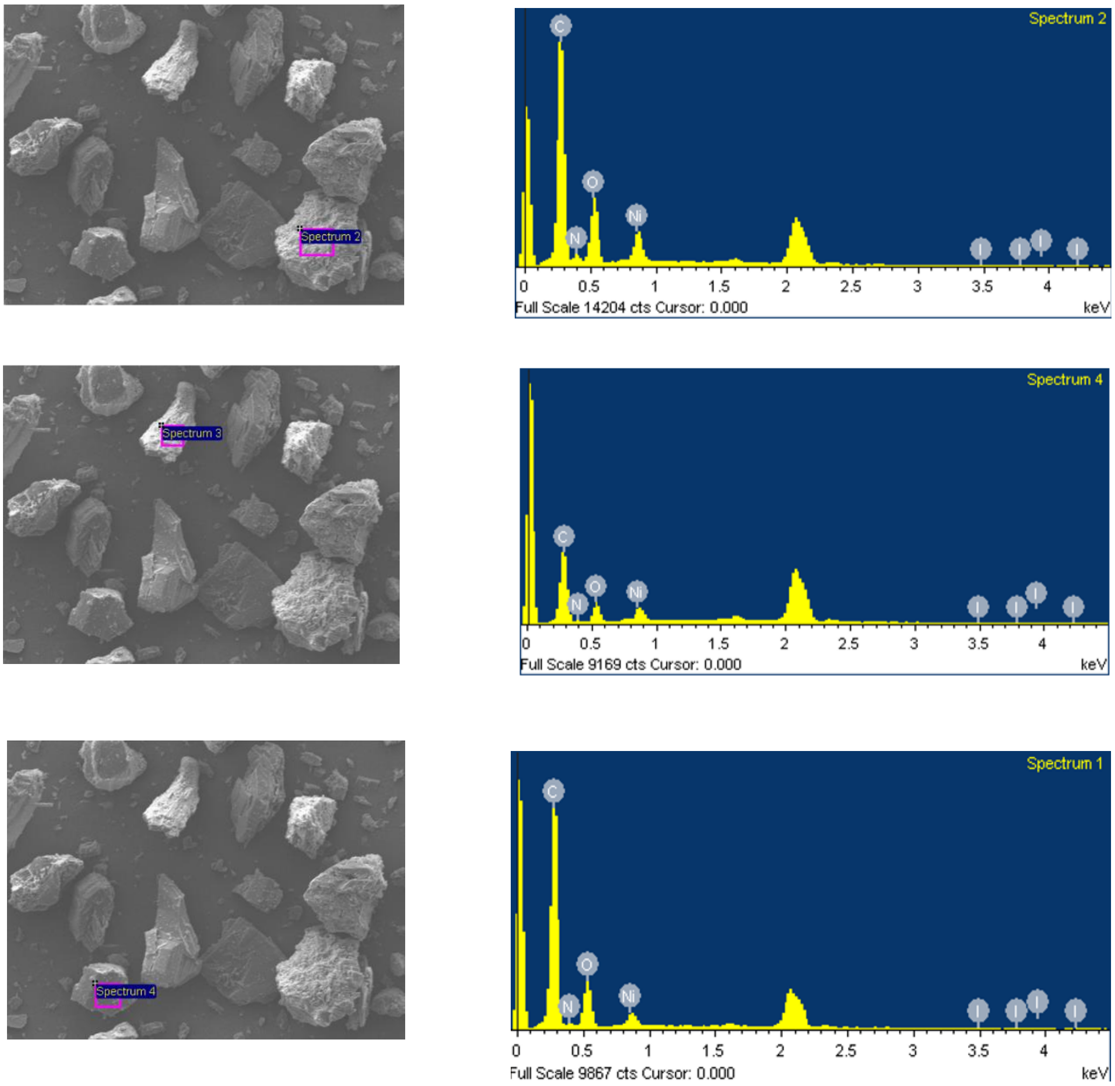

	1st		2nd		3rd	
Element	Weight %	Atomic %	Weight %	Atomic %	Weight %	Atomic %
C K	54.07	64.91	53.37	65.89	63.35	72.98
N K	10.34	10.64	8.40	8.89	4.57	4.50
O K	23.50	21.57	23.23	21.52	23.88	20.61
Ni L	11.62	2.85	14.32	3.62	8.08	1.90
I L	0.46	0.03	0.68	0.08	0.12	0.01
Totals	100.00		100.00		100.00	

Figure S26. EDX data for complex **8** at three different regions.

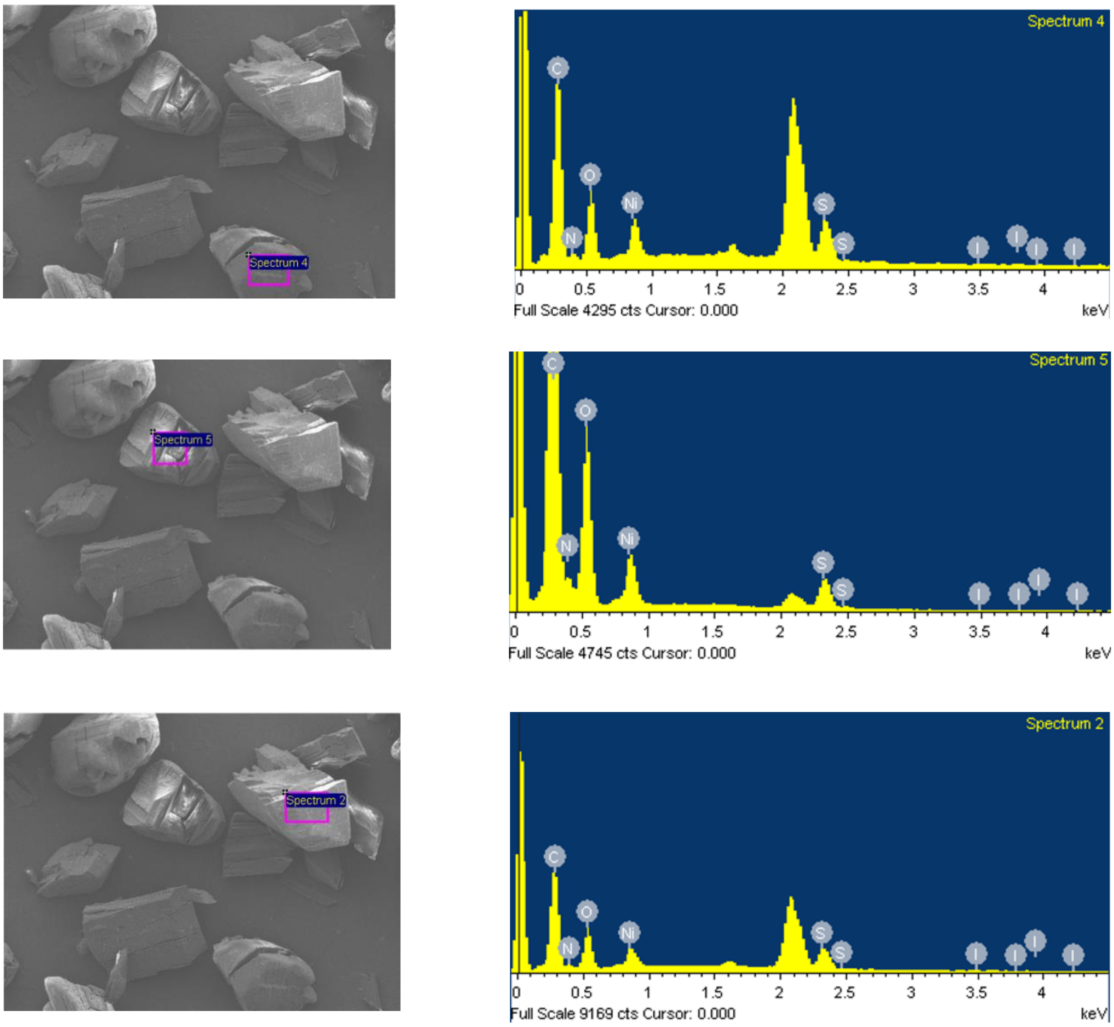

	1st		2nd		3rd	
Element	Weight %	Atomic %	Weight %	Atomic %	Weight %	Atomic %
C K	61.92	72.47	52.46	66.46	52.73	67.47
N K	3.87	3.79	4.86	5.28	4.56	5.01
O K	22.87	20.09	22.48	21.20	20.77	19.95
S K	4.21	1.85	7.65	3.63	8.38	4.02
Ni L	7.12	1.70	12.54	3.25	13.54	3.27
I L	0.01	0.10	0.01	0.18	0.02	0.28
Totals	100.00		100.00		100.00	

Figure S27. EDX data for complex **9** at three different regions.

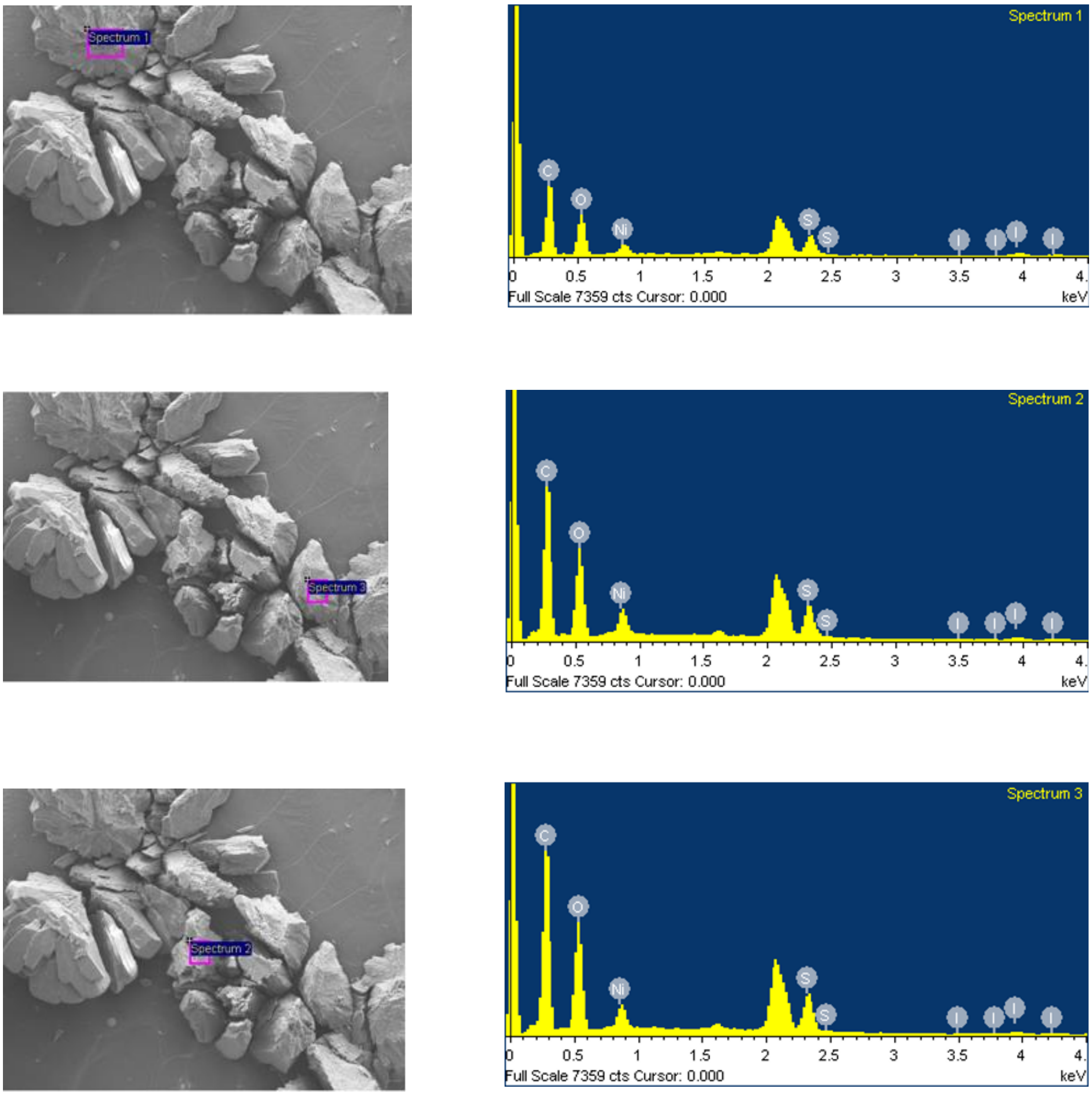

	1st		2nd		3rd	
Element	Weight %	Atomic %	Weight %	Atomic %	Weight %	Atomic %
C K	47.33	66.52	49.98	66.61	51.03	66.20
O K	23.33	24.62	25.87	25.88	27.79	27.06
S K	9.21	4.85	7.59	3.79	7.74	3.76
Ni L	8.63	2.48	11.10	3.03	9.30	2.47
I L	11.50	1.53	5.46	0.69	4.14	0.51
Totals	100.00		100.00		100.00	

Figure S28. Color changes of complex **1** heated at 25 and 60 °C: (a) without heating and heated for (b) 30, (c) 60, (d) 120, (e) 180 and (f) 360 minutes.

25 °C

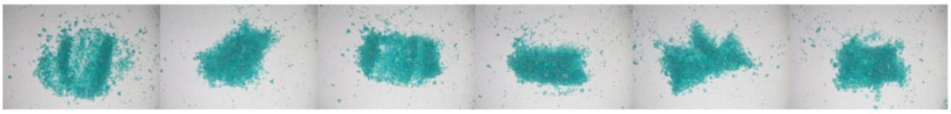

(a) (b) (c) (d) (e) (f)

60 °C.

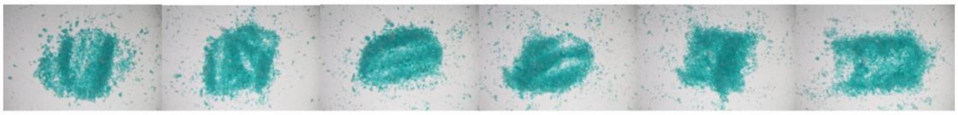

(a) (b) (c) (d) (e) (f)

Figure S29. Color changes of complex **2** heated at 25 and 60 °C: (a) without heating and heated for (b) 30, (c) 60, (d) 120, (e) 180 and (f) 360 minutes.

25 °C

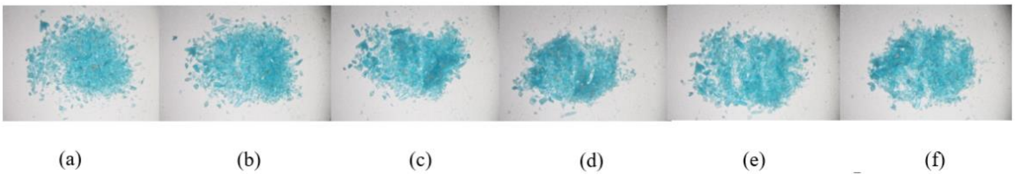

60 °C.

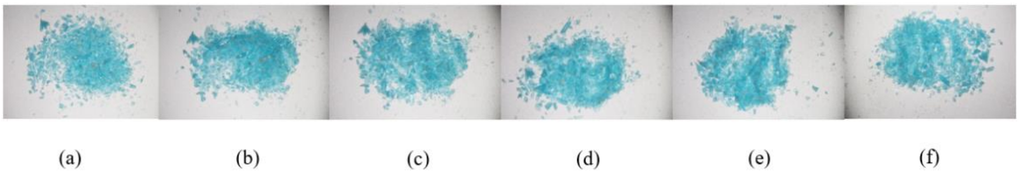

Figure S30. Color changes of complex **3** heated at 25 and 60 ºC: (a) without heating and heated for (b) 30, (c) 60, (d) 120, (e) 180 and (f) 360 minutes.

25 ºC

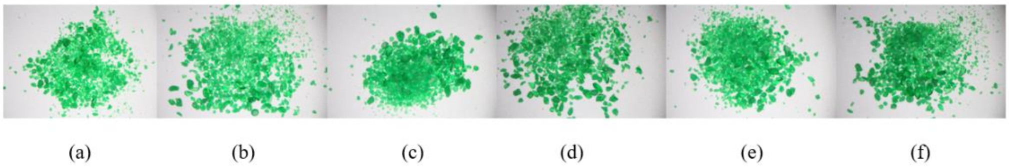

60 °C.

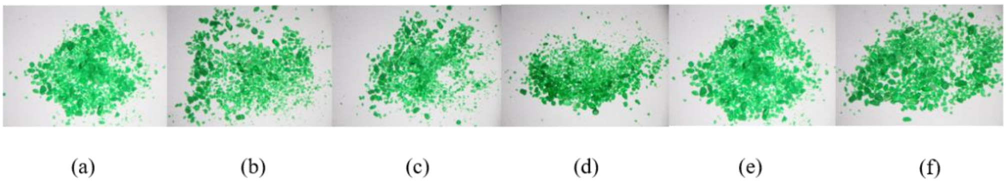

Figure S31. Color changes of complex **4** heated at 25 and 60 ºC: (a) without heating and heated for (b) 30, (c) 60, (d) 120, (e) 180 and (f) 360 minutes.

25 ºC

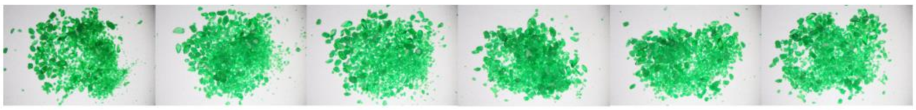

(a) (b) (c) (d) (e) (f)

60 °C.

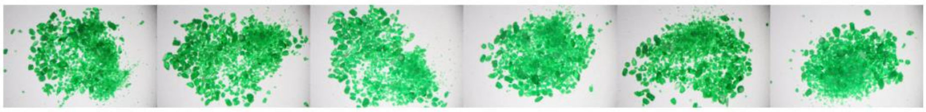

(a) (b) (c) (d) (e) (f)

Figure S32. Color changes of complex **5** heated at 25 and 60 ºC: (a) without heating and heated for (b) 30, (c) 60, (d) 120, (e) 180 and (f) 360 minutes.

25 ºC

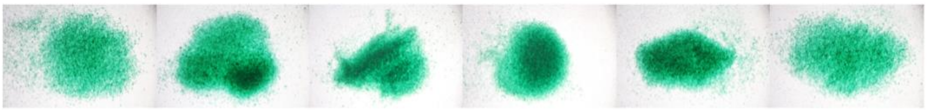

(a) (b) (c) (d) (e) (f)

60 °C.

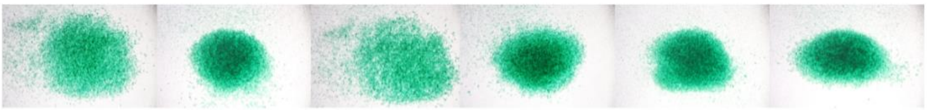

(a) (b) (c) (d) (e) (f)

Figure S33. Color changes of complex **6** heated at 25 and 60 ºC: (a) without heating and heated for (b) 30, (c) 60, (d) 120, (e) 180 and (f) 360 minutes.

25 ºC

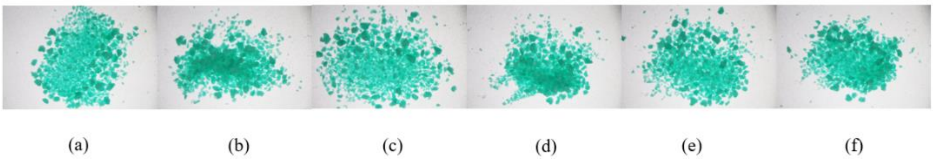

60 °C.

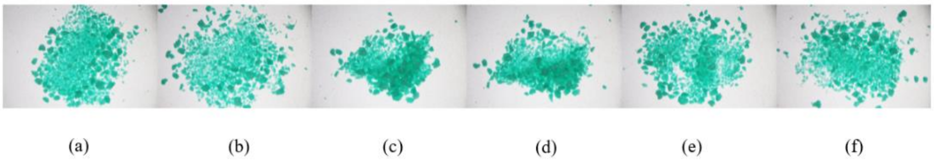

Figure S34. EDX data for iodine-adsorbed complex **1**.

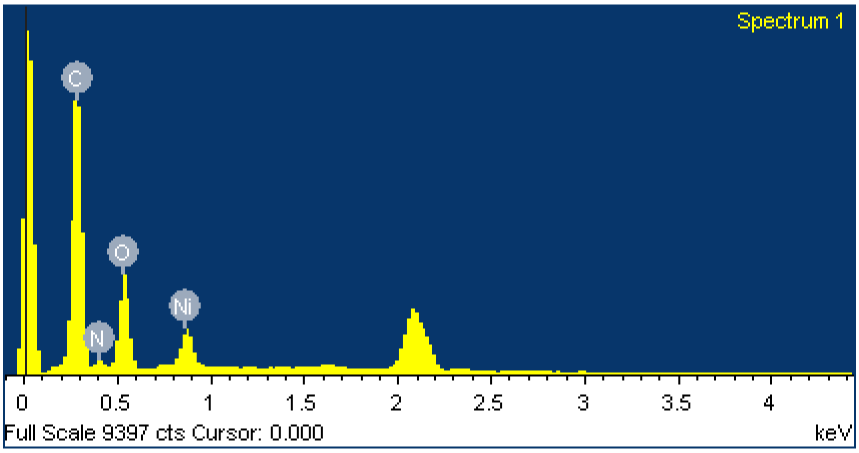

Element	Weight%	Atomic%
C K	52.41	64.68
N K	7.19	7.60
O K	25.99	24.08
Ni L	14.41	3.64
Totals	100.00	

Figure S35. EDX data for iodine-adsorbed complex **2**.

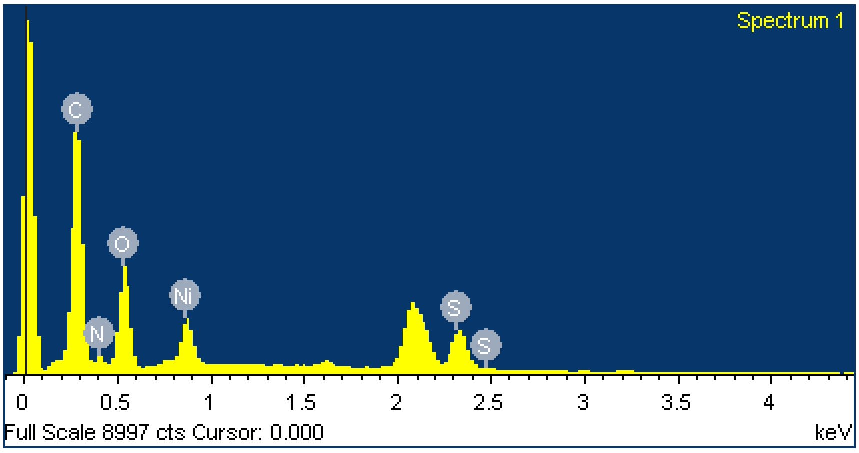

Element	Weight%	Atomic%
C K	49.88	63.50
N K	7.35	8.03
O K	23.04	22.02
S K	6.09	2.90
Ni L	13.64	3.55
Totals	100.00	

Figure S36. EDX data for iodine-adsorbed complex **3**.

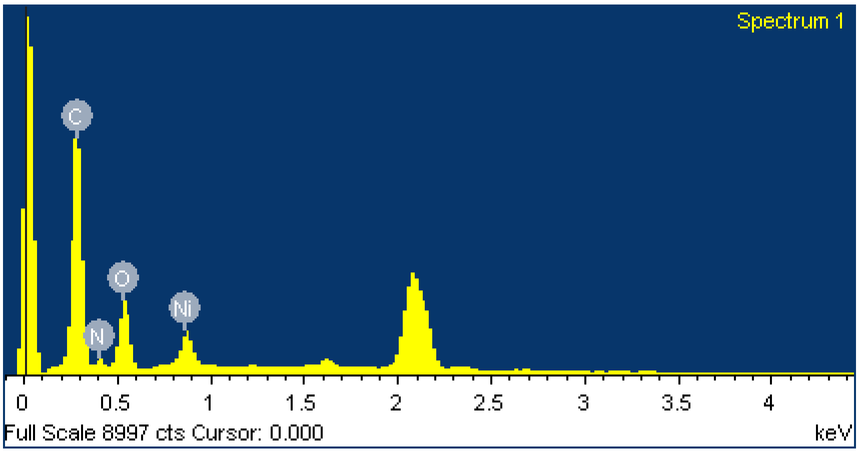

Element	Weight%	Atomic%
C K	52.28	64.75
N K	9.29	9.86
O K	23.14	21.51
Ni L	15.30	3.88
Totals	100.00	

Figure S37. EDX data for iodine-adsorbed complex **4**.

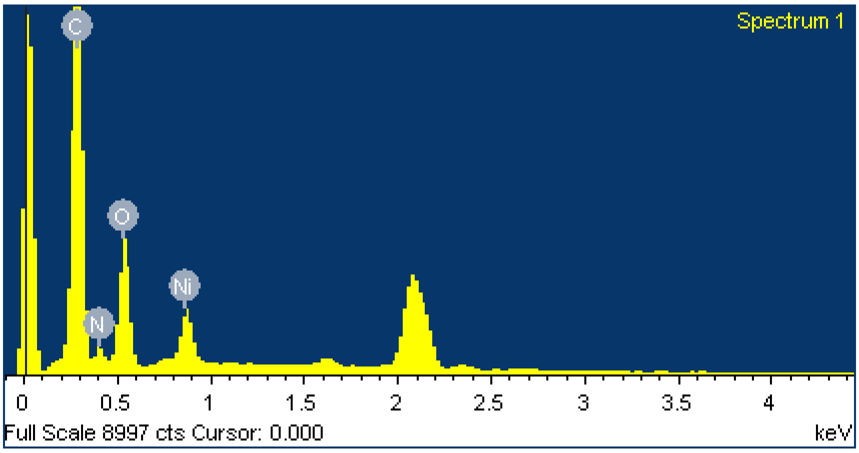

Element	Weight%	Atomic%
C K	52.92	64.62
N K	9.35	9.79
O K	24.25	22.23
Ni L	13.48	3.37
Totals	100.00	

Figure S38. EDX data for iodine-adsorbed complex **5**.

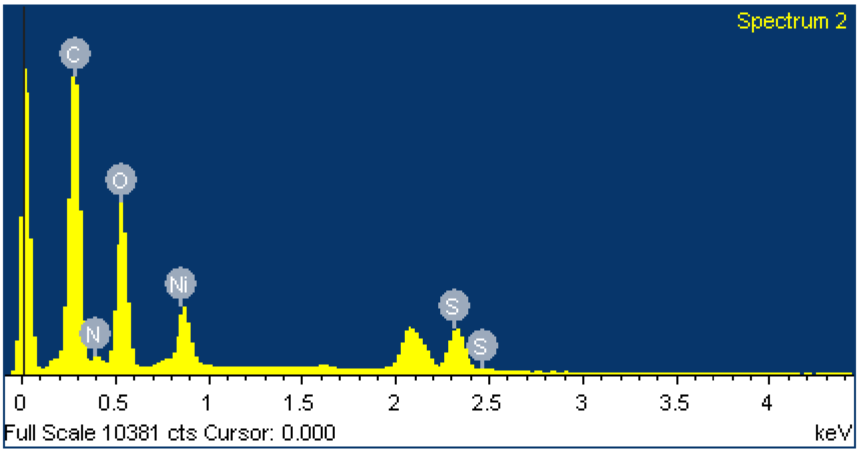

Element	Weight%	Atomic%
C K	47.72	61.13
N K	5.61	6.16
O K	27.67	26.62
S K	5.08	2.44
Ni L	13.92	3.65
Totals	100.00	

Figure S39. EDX data for iodine-adsorbed complex **6**.

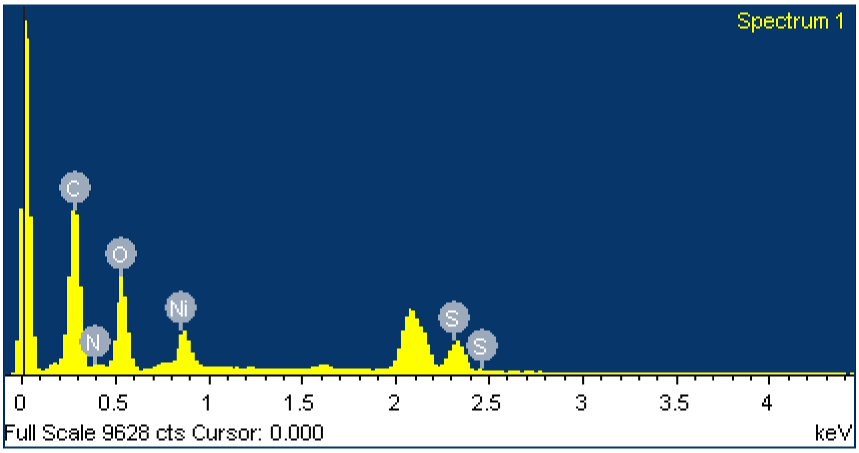

Element	Weight%	Atomic%
C K	48.32	62.53
N K	4.28	4.74
O K	26.50	25.75
S K	6.58	3.19
Ni L	14.33	3.79
Totals	100.00	

Figure S40. The N_2 adsorption for complex **7** at 77 K.

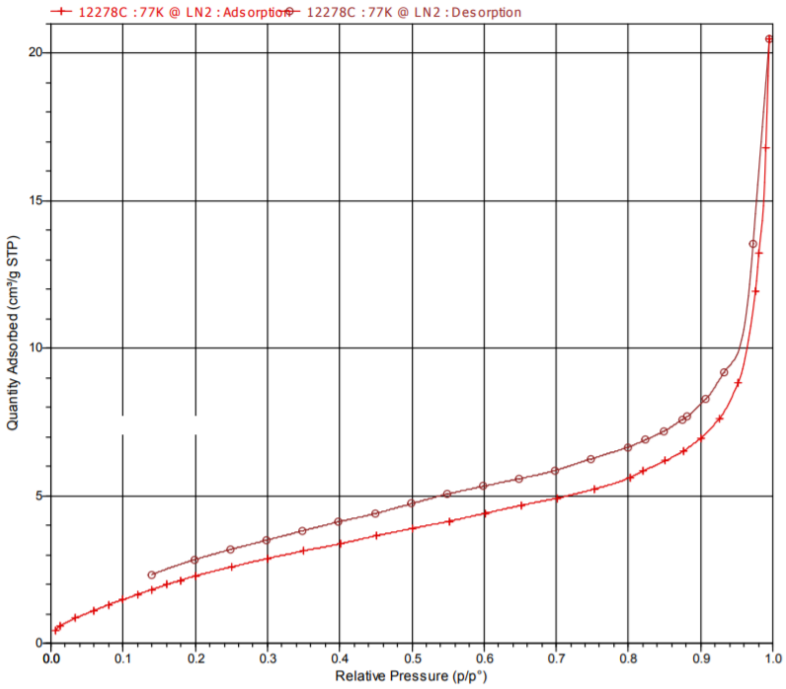

Figure S41. The N_2 adsorption for complex **8** at 77 K.

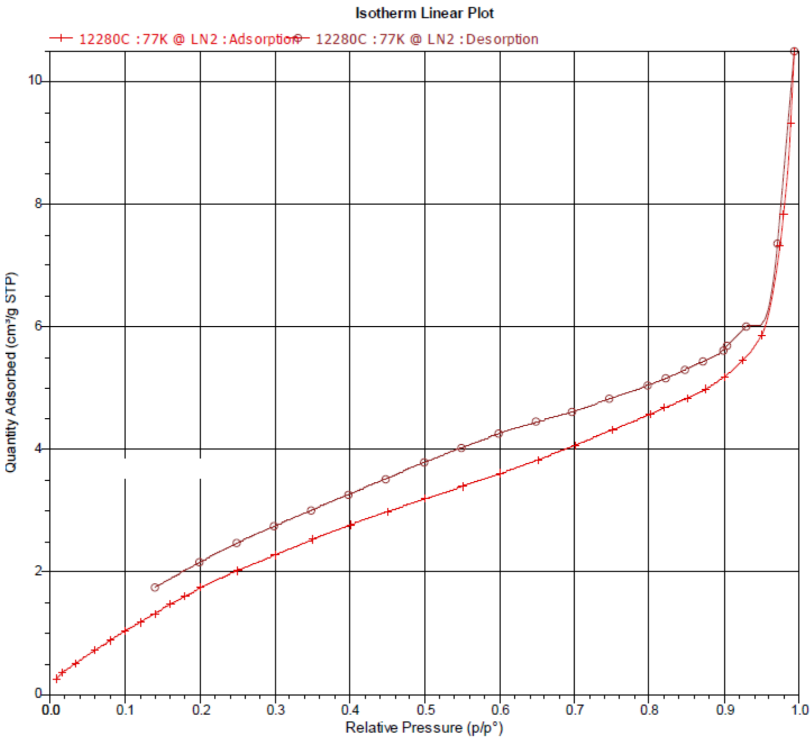

Figure S42. The N_2 adsorption for complex **9** at 77 K.

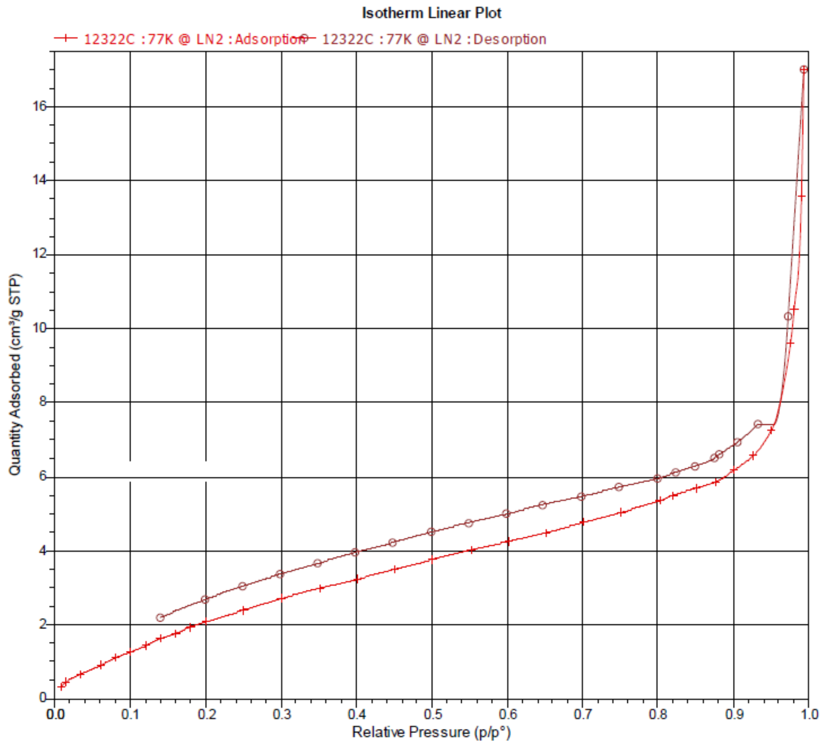

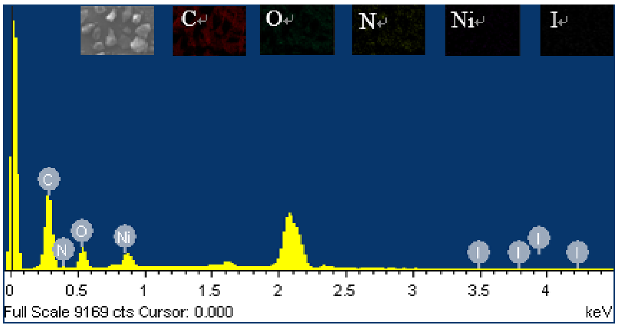
C
O
N
Ni
I
0 0.5 1 1.5 2 2.5 3 3.5 4
Full Scale 9169 cts Cursor: 0.000
keV

(a)

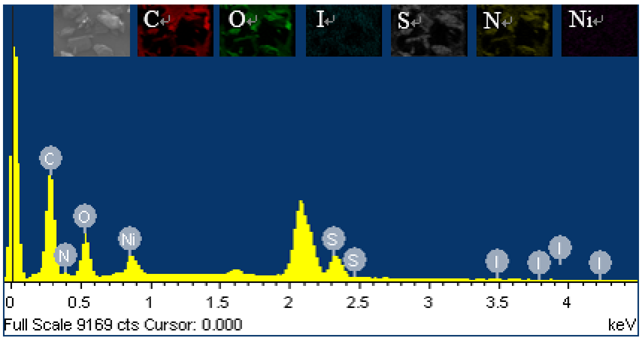
C
O
I
S
N
Ni
0 0.5 1 1.5 2 2.5 3 3.5 4
Full Scale 9169 cts Cursor: 0.000
keV

(b)

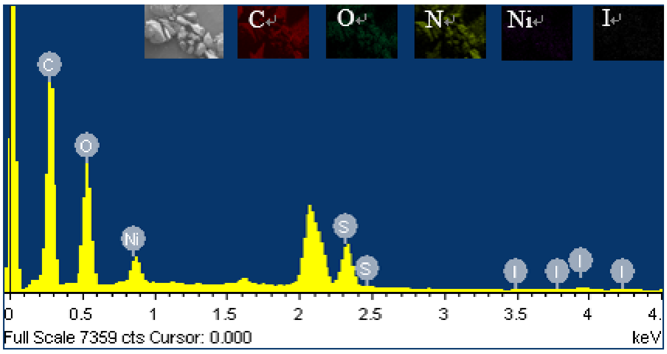
C
O
N
Ni
I
0 0.5 1 1.5 2 2.5 3 3.5 4
Full Scale 7359 cts Cursor: 0.000
keV

Table S7. Solvent accessible volumes of complexes **1** – **9**.

	Solvent assemble volume %
**1**	3.2
**2**	12.6
**3**	10.4
**4**	10.2
**5**	15.2
**6**	15.6
**7**	6.9
**8**	9.3
**9**	13.7
